# Awareness and practice of emergency contraception at a private university in Nigeria

**DOI:** 10.1186/s13104-015-1204-y

**Published:** 2015-06-04

**Authors:** Jacob Olumuyiwa Awoleke, Abiodun Idowu Adanikin, Adeola Awoleke, Moyinoluwa Odanye

**Affiliations:** Department of Obstetrics and Gynaecology, Ekiti State University Teaching Hospital, Ado-Ekiti, Nigeria; School of Nursing and Midwifery, Ekiti State University Teaching Hospital, Ado-Ekiti, Nigeria

**Keywords:** Africa, Student, Undergraduate, Private university, Emergency contraception

## Abstract

**Background:**

The pursuit of formal education now causes many people in developing countries to marry later in life, thereby leading to increased premarital sex and unintended pregnancies. Efforts have been made to characterize awareness and use of emergency contraception (EC) among undergraduate students in public universities in Nigeria; however, it is not known if students in private tertiary institutions adopt different practices or if having an affluent family background plays a role. This pilot study therefore aimed to assess the awareness and use of EC among students at a private Nigerian university toward assisting education planners in developing strategies in improving students’ reproductive well-being.

**Results:**

Out of 94 female students, 42 (44.7%) had sexual experience, but only 32 (34.0%) were currently sexually active. Six students (6.4%) had had unwanted pregnancies, of which all but one were terminated. Fifty-seven respondents (60.6%) were aware of EC, though only 10 (10.6%) ever practiced it. The greatest source of EC information was from health workers and peers; the lowest source was family or relatives. Most respondents desired orientation and availability of EC on campus. EC awareness among the students was predicted by upper social class background (adjusted odds ratio [OR], 2.73; 95% confidence interval [CI], 1.06–7.45) and upbringing in the Federal Capital Territory (adjusted OR, 4.45; 95% CI, 1.56–14.22).

**Conclusions:**

Though awareness of EC was higher among the private university students in this study than at most public universities, there was no difference in EC usage. A high pregnancy termination rate was observed; dilatation and curettage were mainly adopted. In Nigeria, youth-friendly reproductive health information and access should not be limited to government-owned tertiary institutions but also extended to private ones.

## Background

Emergency contraception (EC) refers to a group of birth control modalities that when used after unprotected intercourse within defined time limits can prevent an unwanted pregnancy [1]. Unlike regular methods of contraception adopted before sexual intercourse, EC is used to avoid pregnancy after unprotected sex. It therefore has a last-resort potential of avoiding unwanted pregnancy and, by extension, unsafe abortion, which is a desirable goal—especially in countries where abortion is illegal [2]. Research has shown that EC is largely underutilized worldwide, and it has been referred to as one of the best-kept secrets in reproductive health [3].

Approximately 6.8 million pregnancies occur annually in Nigeria; the majority of these pregnancies are unplanned and usually unwanted [4]. More than 610,000 induced abortions occur annually in the country, and young people are responsible for over 60% of them [5]. When pregnancies are unwanted and unplanned in many low-income countries, they have a negative impact on the reproductive health of young adults since some of them resort to unsafe abortions, which are often complicated by genital infections, infertility, permanent morbidities, and sometimes death [2, 5–7].

About 44% of the Nigerian population consists of young people [8]. In contrast to previous practice, many Nigerians are now marrying later because of longer formal education, and they are increasingly engaging in premarital sex [9]. Therefore, the need for EC has grown in this category of individuals, who engage in sporadic, erratic, casual sexual intercourse, since EC offers a valuable means of preventing pregnancy [2]. Depending on the method used, EC can reduce the risk of becoming pregnant from a single act of sexual intercourse by 75–99% [10].

Some studies have examined the awareness and practice of EC among undergraduate students in Nigeria, but none has considered its practice among private university students. Privatization of the university system is a relatively recent development in this country. Because of the exorbitant fees, the majority of students attending private universities are from the upper socioeconomic class, who may be better informed about EC. Another consideration is that although their social background may assist in enhancing their academic progress, their younger age within the university system could make them vulnerable to more sexual experimentation. The present study aimed to survey sexual practices, EC awareness, and EC use in Nigerian private university students. We believed that the results would shed light on some gray areas in EC practices and assist education planners in crafting strategies toward improving students’ reproductive well-being.

## Methods

This pilot cross-sectional study about EC awareness and practices among female undergraduates at a private university was conducted in southwest Nigeria between July and August 2013. The Ethics Committee of Ekiti State University Teaching Hospital, Ado-Ekiti, Nigeria reviewed the study protocol and gave its approval. Study permission was also obtained from the authority of the private university in question, though it requested its name not be mentioned.

The primary outcome of interest was the use of EC among the students. We employed a previous report about the overall prevalence of EC being 6% (30/500) among undergraduates in Nigeria to calculate the minimum sample size required [11]. We determined that at least 87 participants would be needed for a standard normal deviation of 1.96 at the 95% confidence level with an error margin of 0.05. We administered 100 questionnaires to allow for non-response.

The private university studied has six faculties: law; engineering; medicine and health sciences; social and management studies; social justice; and natural and applied sciences. We used a multistage sampling approach to select the respondents. We first chose two of the faculties (engineering; social and management sciences) by balloting. We then selected two departments from each of those faculties also by balloting. They were the departments of Civil and Mechanical Engineering from the Faculty of Engineering and the departments of Psychology and Economics from the Faculty of Social and Management Sciences. We subsequently used a simple random sampling technique in administering 25 questionnaires to female students in each of the four departments.

Before conducting the study, we pre-tested a semi-structured questionnaire at the Federal Polytechnic, Ado-Ekiti, which is a government tertiary institution in the same locality, using ten respondents. We did this to assess the ambiguity and comprehensibility of the study instrument. We subsequently modified any ambiguous questions for ease of comprehension.

After obtaining informed written consent from the participants, the questionnaires were distributed to them by hand. Respondents were given time to complete the questionnaires, which were retrieved the same day to avoid losses. The net response rate was 94%, and we based our analysis on that.

The data obtained were structured with respect to the sociodemographic characteristics of the respondents, and they included the following: age; religion; social class of the parents; place of upbringing; relationship status; year of study at the institution; sexual practices; occurrence of unwanted pregnancy and its outcome; awareness and practice of EC; and desire for EC education in the school’s orientation program. The collected data were entered into SPSS package version 17 [SPSS Inc., Chicago, USA] and analyzed. The results were expressed as simple percentages, and logistic regression analysis was employed as appropriate. We set the level of significance (α) at 0.05.

## Results

Table 1 shows the sociodemographic characteristics of the students. Only two (2.1%) were married; 11 (11.7%) cohabited with their boyfriends during holidays; the others were single. The majority—71 students (75.6%)—were from families in the upper social strata (Social Class I and II); 57 (60.6%) were originally from southwestern Nigeria.

**Table 1:** Sociodemographic characteristics of respondents

Variables	Frequency (%)	Range
Age (years)		16–25
≤22	69 (73.4)	
≥23	25 (26.6)	
Religion		
Christianity	80 (85.1)	
Islam	14 (14.8)	
Family social class		
Class I	31 (33.0)	
Class II	40 (42.6)	
Class III	14 (14.9)	
Class IV	9 (9.5)	
Class V	0 (0)	
Place of childhood upbringing *(Nigerian region)*		
Southwest	57 (60.6)	
Southeast	4 (4.3)	
South-south	8 (8.5)	
Northeast	2 (2.1)	
Northwest	3 (3.2)	
North central	2 (2.1)	
FCT	18 (19.2)	
Relationship status		
Single	81 (86.1)	
Cohabits with boyfriend	11 (11.7)	
Married	2 (2.1)	
Year of study		
1st year	7 (7.4)	
2nd year	23 (24.5)	
3rd year	36 (38.3)	
4th year	17 (18.1)	
5th year	11 (11.7)	

With respect to sexual practices, 42 (44.7%) had sexual experience (Table 2). Among those sexually exposed, 33 (78.6%) had their sexual debut at or before the age of 22 years; 27 respondents (64.3%) with sexual experience used contraceptive protection. In the sampled population, 32 respondents (34.0%) were currently sexually active (had had sex within the previous 6 months), and six (6.4%) had had an unwanted pregnancy, though only one (16.7%) resulted in birth. The most practiced form of pregnancy termination was dilatation and curettage.

**Table 2:** Sexual practices of respondents

Variable	Frequency (%)
Ever had sex, *n* = 94	
Yes	42 (44.7)
No	52 (55.3)
Age at sexual debut (years), *n* = 42	
≤22	33 (78.6)
≥23	9 (21.4)
Was exposure protected?, *n* = 42	
Yes	27 (64.3)
No	15 (35.7)
Presently sexual active?, *n* = 94	
Yes	32 (34.0)
No	62 (66.0)
Had unwanted pregnancy?, *n* = 94	
Yes	6 (6.4)
No	88 (93.6)
Outcome of unwanted pregnancy, *n* = 6	
Delivered	1 (16.7)
Aborted	5 (83.3)
Abortion method, n = 5	
Medical	2 (40.0)
D&C	3 (60.0)

Table 3 presents the knowledge and practice of EC among the respondents. Over half of the respondents—57 (60.6%)—were aware of EC, but only 10 (10.6%) ever practiced it. With regard to sources of information, peers and health workers ranked highest; the lowest source was family or relatives. The levonorgestrel EC pill (Postinor) was the most commonly adopted method (seven respondents; 70.0%), followed by Menstrogen (two; 20.0%), and intrauterine contraceptive device (one; 10.0%). Twelve respondents (21.0%) believed that EC was effective before sex; 13 (22.8%) had no knowledge of the period of its effectiveness. Twenty students (35.1%) understood that its effectiveness was confined to within 24 h after intercourse, whereas 11 respondents (19.3%) declared that it was still effective until 72 h after sex. Just one student believed that it could be used up to 5 days post-intercourse.

**Table 3:** Knowledge and practice of emergency contraception (EC) among respondents

Variable	Frequency (%)
Aware of EC, n = 94	
Yes	57 (60.6)
No	37 (39.4)
Source of EC knowledge, *n* = 57	
Media	12 (21.0)
Friends/peers	14 (24.6)
Health worker	17 (29.8)
Family/relatives	1 (1.8)
Books/magazine	9 (15.8)
Campaign outreaches	4 (7.0)
Ever used EC, *n* = 94	
Yes	10 (10.6)
No	84 (89.4)
Type of EC used, *n* = 10	
LNG pill (Postinor)	7 (70.0)
Menstrogen	2 (20.0)
IUCD	1 (10.0)
Source of EC procurement, *n* = 10	
Public hospital	1 (10.0)
Private hospital	4 (40.0)
Pharmacy/chemist	5 (50.0)
When is EC effective?, *n* = 57	
Before sex	12 (21.0)
Within 24 h after sex	20 (35.1)
Within 72 h after sex	11 (19.3)
Up to 5 days after sex	1 (1.8)
Don’t know	13 (22.8)
Perceived side effect of EC, *n* = 57	
Nausea and vomiting	4 (7.0)
Weight gain	7 (12.3)
Menstrual irregularities	17 (29.8)
Infertility	16 (28.1)
Bleeding disturbances	4 (7.0)
Not known	9 (15.8)
Desire EC orientation on campus, *n* = 94	
Yes	55 (58.5)
No	23 (24.5)
No response	16 (17.0)
Desire EC is available on campus, *n* = 94	
Yes	48 (51.1)
No	30 (31.9)
No response	16 (17.0)
Will use EC in future, *n* = 94	
Yes	44 (46.8)
No	30 (31.9)
No response	20 (21.3)

*LNG* levonorgestrel.

Most respondents (55 students; 58.5%) desired EC orientation on campus; 48 students (51.1%) wanted EC to be available on campus. Forty-four students (46.8%) stated that they would use EC in future. Our regression model showed that being from an upper social class (adjusted odds ratio [OR], 2.73; 95% confidence interval [CI], 1.06–7.45) and having an upbringing in the Federal Capital Territory (adjusted OR, 4.45; 95% CI, 1.56–14.22) predicted EC awareness among the students (Table 4).

**Table 4:** Regression analysis of predictors of emergency contraception (EC) awareness

Variable	EC awareness *n* (%)		Total *n* (%)	Adjusted odd ratio	95% CI
	Yes	No			
Age (years)					
≤22	38 (55.1)	31 (44.9)	69 (100)	1.00	
≥23	19 (76.0)	6 (24.0)	25 (100)	0.41	0.14–1.07
Religion					
Christianity	50 (62.5)	30 (37.5)	80 (100)	1.00	
Islam	7 (50.0)	7 (50.0)	14 (100)	1.66	0.55–5.03
Social class					
I	15 (48.4)	16 (51.6)	31 (100)	2.73	1.06–7.45*
II	29 (72.5)	11 (27.5)	40 (100)	1.00	
III	6 (42.9)	8 (57.1)	14 (100)	3.35	1.03–12.01*
IV	7 (77.8)	2 (22.2)	9 (100)	0.86	0.15–3.85
Place of childhood upbringing					
Southwest	40 (70.2)	17 (29.8)	57 (100)	1.00	
Southeast	4 (100)	0 (0)	4 (100)	0.26	0.02–3.72
Southsouth	3 (37.5)	5 (62.5)	8 (100)	3.64	0.92–16.76
Northcentral	0 (0)	2 (100)	2 (100)	11.57	0.32–1.00
Northeast	2 (100)	0 (0)	2 (100)	0.46	0.01–13.31
Northwest	2 (66.7)	1 (33.3)	3 (100)	1.39	0.15–9.04
FCT	6 (33.3)	12 (66.7)	18 (100)	4.45	1.56–14.22*
Relationship status					
Single	47 (58.0)	34 (42.0)	81 (100)	1.00	
Cohabit with boyfriend	8 (72.7)	3 (27.3)	11 (100)	0.57	0.14–1.98
Married	2 (100)	0 (0)	2 (100)	0.28	0.01–7.68
Year of study					
1st year	2 (28.6)	5 (71.4)	7 (100)	5.55	0.85–74.96
2nd year	13 (56.5)	10 (43.5)	23 (100)	1.96	0.68–5.89
3rd year	26 (72.2)	10 (27.8)	36 (100)	1.00	
4th year	10 (58.8)	7 (41.2)	17 (100)	1.80	0.56–5.93
5th year	6 (54.5)	5 (45.5)	11 (100)	2.14	0.57–8.28

* Statistically significant.

## Discussion

Unlike most previous studies, which considered subjects in government-owned institutions, the present investigation examined the awareness and practice of EC in a private tertiary institution in Nigeria. We found close to half of our respondents had had sexual intercourse, and a third were currently sexually active. The awareness of EC was above average among the students, but its practice was low. Consequently, unwanted pregnancies occurred, almost all of which were terminated.

When compared with sexual practices of female students in government-owned tertiary institutions in Nigeria, the proportion (44.7%) of respondents with sexual experience in the present study was close to the 48.2% reported for the University of Ibadan but significantly lower than the 85.3% of the University of Portharcourt [12, 13]. In our study, the proportion (34.0%) of students who were currently sexually active was also less than the 43% observed at the University of Benin and the 56.1% at the University of Lagos [5, 14]. This observation controverts the assumed likelihood of more sexual experimentation among these “privileged” students.

Our finding of 60.6% EC awareness among the private university students is similar to the value reported for public institutions in Lagos and Benin, Nigeria where 58 and 67.8% of female students had heard of EC [5, 14]. It is, however, higher than the awareness of 24.3, 38.1, and 46%, respectively, from public universities in the Nigerian states of Oyo, Anambra, and Ebonyi [11, 12, 15]. A comparison with other African universities shows that EC awareness is higher than the 51.4% reported for Ghana and 45.1% for Uganda [16, 17], though it is much lower than the 95% reported for Princeton University and Mexico [18, 19].

The practice of EC by the students in the present study showed no remarkable departure from observations in public tertiary institutions in Nigeria [11, 12, 15]. Unlike in those public universities, the private students mostly obtained EC awareness from health workers and then from peers. It is likely that the high socioeconomic background of our respondents offered them access to health facilities and personnel, which provided reproductive health services. Further, being from an upper social class and having an upbringing in the Federal Capital Territory led to increased EC awareness.

We found that 20% of respondents erroneously used Menstrogen as EC. Only a fifth of students knew the correct timing of EC usage. This is in line with reports from sister public universities [14, 20]. It is possible that distorted information about EC was obtained from such sources as peers and magazines or other media. This finding points to the need for greater education on reproductive health in general and EC in particular. Specifically, establishing youth-friendly reproductive health centers at each university would be an appropriate move.

Owing to its attendant complications, the dominance of pregnancy termination by dilatation and curettage indicates both a short- and long-term danger for the students in this study. In an environment where abortion is not legalized, a more sensible precautionary measure would be to increase EC awareness among young people. This need certainly exists, as evidenced by more than half of our respondents wanting campus orientation about EC and EC availability on campus.

One strength of the present investigation is its pilot nature: no previous study in Nigeria has examined EC usage at a private university. The small sample size could limit the generalizability of our results; however, the fact that the sample size was calculated using a standard formula and sampling bias was minimized gives the study robustness and reproducibility. We recognized from the outset that the subject of our research was unlikely to receive ready acceptance by the authorities of most private educational institutions, which are intent on preserving their moral reputation.

## Conclusions

Contrary to expectations, we found the prevalence of sexual activity among private university female students to be slightly lower than that of their counterparts in public universities in Nigeria. Higher socioeconomic background and place of childhood upbringing were found to be predictors of EC awareness. However, we observed that the actual use of EC did not differ from that among other female students in public tertiary institutions. The very high pregnancy termination rate of 84% among those who became pregnant underlines the need for effective contraceptive education on campuses; it is necessary as a preventive measure in a society where abortion is illegal and unsafe termination through dilatation and curettage still dominates. We also recognize that educating young people about safer pregnancy termination methods may be needful, though Nigeria’s current restrictive abortion laws present a stumbling block. It is necessary to make concerted efforts to promote youth-friendly reproductive health services and rights at both public and private institutions.

## Abbreviation

EC: emergency contraception
